# A Quantitative Spectroscopic Study of the Bleaching Phenomena in Plasticized Formulations Containing PVC Exposed to Outdoor Conditions

**DOI:** 10.3390/polym11091481

**Published:** 2019-09-11

**Authors:** Elizabeth González-Falcón, Martin Arellano, M. Judith Sanchez-Peña, L. Javier González-Ortiz

**Affiliations:** 1 Chemistry Department, Universidad de Guadalajara, 44100 Guadalajara, Mexico; elizabeth_glezf@yahoo.com.mx (E.G.-F.); judithsan1@yahoo.com.mx (M.J.S.-P.); 2 Chemical Engineering Department, Universidad de Guadalajara, 44100 Guadalajara, Mexico; marellan@cencar.udg.mx

**Keywords:** plasticized PVC, bleaching phenomena, outdoor conditions, degrading behavior, polyene accumulation behavior, semi-empirical model

## Abstract

In this work, a quantitative spectroscopic study of the bleaching phenomena occurring in plasticized formulations containing poly(vinyl chloride) was performed, proposing a general methodology to comparatively analyze the effect of degrading conditions on the polyene accumulation behaviors (PABs) exhibited by a set of tested formulations. In the study, a set of environmental indexes (temperature (T*), UV energy (UV*), and days with rain) were proposed, which allowed for the suitable globalization of the changing environmental conditions occurring throughout the different degrading periods. A procedure to numerically describe the PAB, followed by each formulation undergoing each degrading condition was also proposed, which required only two primary fitting parameters and four secondary fitting parameters. Then, the combined effects of certain environmental conditions on the PABs were studied, quantifying the stabilizing effects of the rain and the combined decrement on the T* and UV* indexes. Finally, on the basis of the proposed fitting equation and the values of its fitting parameters, the relative importance of the dehydrochlorination reactions as compared with the photo-oxidative reactions simultaneously occurring in the studied systems was estimated.

## 1. Introduction

Because of its favorable balance cost/performance, poly(vinyl chloride) (PVC) is one of the three most frequently-used conventional plastic materials, alongside polypropylene and polyethylene [1,2]. The type industrial formulations containing PVC that are exposed at outdoor conditions usually undergo undesirable effects, which are strongly dependent on the specific environmental conditions (e.g., the oxygen [3,4,5,6] or other aggressive compounds present [3,4,6], humidity [1,3,4,6,7], and sunlight exposure [1,5,7,8]), but also depending on the structural defects produced in the polymer chain during its synthesis procedure (e.g., long and short branches [7,8], unsaturation internal or terminal [7], internal allylic chloride [1], tertiary chloride [1,9,10], chain end groups with initiator residues [10], unsaturated end groups [10], or molecules/chemical groups oxygenated [1,10]).

To avoid/reduce the degradation level during the processing of such formulations, as well as to improve their properties during their useful lives, formulations containing PVC usually include stabilizing agents (e.g., calcium stearate (–CaSt_2_–) and/or zinc stearate (–ZnSt_2_–) [11,12]) and, eventually, coestabilizers (e.g., pentaerythritol [13,14,15], sorbitol [16], β-diketone [17,18]). It is well known that ZnSt_2_ prevents the initial dehydrochlorination of PVC chains, but as a product of its stabilizing action, ZnCl_2_ is produced [19,20]. Unfortunately, when ZnCl_2_ is accumulated in the system at a certain level, it is able to promote a sudden dehydrochlorination of the PVC chains [19,20,21]. Aside from this, CaSt_2_ mainly reacts with the HCl produced by dehydrochlorination reactions, avoiding, in this way, the auto-accelerating action of this acid [19]. Nevertheless, it is well recognized that the use of Ca/Zn stearate mixtures involves a synergistic stabilizing action [19,20,21], which has mainly been attributed to the reaction between ZnCl_2_ and the CaSt_2_, where CaCl_2_ (an inert) and ZnSt_2_ (a stabilizer) are formed.

During outdoor weathering, the main degradation reactions occurring in PVC formulations are produced by the combined effect of the oxygen presence and light radiation (UV and visible); as a whole, this process can be technically named photo-thermal oxidation. During photo-thermal oxidation, the production of polyenes and their subsequent oxidation seems to occur simultaneously, thus arising, from the chemical point of view, two competing processes [22]. The first process is the formation of large conjugated polyene sequences on the PVC chain (zipper dehydrochlorination); these reactions are, at least partially, responsible for the color change of the irradiated material [1,22,23,24,25,26]. As additional information, it has been reported that, in the presence of oxygen, such dehydrochlorination is significantly accelerated [5]. Complementarily, the second process is the oxidation of these large chains of conjugated double bonds, which are readily photo-oxidized in the presence of molecular oxygen, causing the bleaching phenomenon as a collateral effect [1,5,10,27,28,29,30,31]. On the other hand, it has been reported that [5,24] the degradation process can also produce superficial microcracks (especially in non-plasticized samples), which would modify the light diffusion pattern through its thickness and, under certain circumstances, could produce perceptible modifications on the material visual appearance. Unfortunately, although the bleaching phenomenon is known [1,5,10,27,28,29,30,31], there is an absence of quantitative information about its relative importance on the global degradation process, especially in PVC plasticized formulations, with several relevant questions remaining unsolved.

It is important note that the combined effect of oxygen presence and the exposition to ultraviolet light can produce attacks on polyene sequences, but this can also occur on other sites in the polymer chain [30]. However, the bleaching effect occurs when the polyene sequences are attacked, breaking down the conjugated polyene sequences [5,10] and decreasing the concentration of the different polyene sequences. Note that the color production level inherent in the presence of a large conjugated polyene sequence is higher than the one produced by the two (or more) shorter conjugated polyene sequences produced by its breakdown during a given photo-oxidization process [10,28,31].

Several theoretical proposals relative to the mechanism of photo-thermal oxidation of PVC have been reported [22,23,24,25,29], which are only partially complementary. Therefore, as a part of this work, such reactions were systematically summarized, eliminating the equivalent reactions and grouping, when it was chemically suitable, two or more reactions in a single one. The summarized set of reactions is presented in a matrix form in Table 1 and Table 2.

Table 1 presents the reactions affecting the evolution of the concentration of the different polyenes as a function of the degradation time (from now on named: “polyene accumulation behavior”; PAB) and, to simplify their comprehension, they have been grouped as follows: (a) reactions forming polyenes (“polyene formation”, PF), (b) reactions increasing the length of the polyene sequences (“polyenic increasing”, PI), (c) reactions decreasing the length of the polyene sequences (“polyene decreasing”, PD), and (d) other reactions consuming or producing species involved in the previous groups of reactions (other reactions, OR). In addition, Table 2 presents the reactions that simultaneously affect the PAB and the molecular weight distribution on the studied systems.

On the other hand, when performing a comparative analysis of the effect of the degradation conditions on a set of samples using controlled conditions (e.g., at isothermal accelerated weathering experiments, with predefined profiles of humidity and/or light irradiation), is evident when the value(s) of one or more factors were increased or decreased. However, in experiments performed in outdoor conditions (such as those required to characterize the bleaching phenomenon), where the temperature, the solar irradiation and, the environmental humidity are continuously changing, and each parameter follow a different profile with a shape more or less capricious, perform comparative studies is not an easy task. In these circumstances, it is desirable to be able to have a systematic methodology to define globalizing indexes for the environmental conditions that allow performing suitable comparisons.

On the basis of this, the aims of this work are (a) to propose a systematic procedure to quantitatively describe the bleaching phenomenon on industrial grade formulations containing PVC, including the procedures required to establish representative environmental indexes that can be suitably correlated with the individual or combined degrading effects produced by UV irradiation, the environmental temperature profile, and rain presence; (b) to propose procedures to comparatively analyze the PAB in preselected PVC formulations, numerically establishing “master curves”, where the fitting parameters can be used to perform such analysis; and (c) to study, as much as the environmental conditions allow, the individual or coupled degrading effects produced by UV irradiation, temperature, and rain, in natural outdoor conditions.

## 2. Materials and Methods

### 2.1. Materials

To prepare the formulations, the following substances were used: (a) PVC resin from Mexichem Resinas Vinílicas S.A. de C.V., Guadalajara, Mexico (PRIMEX G-30; K-Fikenstcher value of 70), (b) di(2-ethylhexyl) phthalate (DEHP; purity: 99.7%) purchased from Proveedor Químico de Guadalajara S.A. de C.V. (PQG), Guadalajara, Mexico, (c) epoxidized soybean oil (ESO; acidity value: 0.56 mg KOH/g, iodine value: 1.72%) supplied by PQG, (d) calcium stearate (CaO assay: 9.0–10.5%) purchased from Jalmek, Guadalajara, Mexico, and (e) zinc stearate (ZnO assay: 12.5–14%) acquired from Jalmek, Guadalajara, Mexico.

### 2.2. Sample Preparation

The 2 kg batches of PVC compounds were prepared as follows: (a) manually premixing the required amount of stearates (the relative content of stearates for each formulation is presented in Table 3), (b) dry-blending the components (using a 10 L blender, “Tornado”; total blending time: 13 min [11]; the relative content of the used components to prepare each formulation is shown in Table 3), (c) pelletizing the dry-blend using a twin-screw extruder (Leistritz 276L/32D; 125–170 °C, gradually increasing the temperature from feed to die), (d) extruding the pellets to obtain ribbon geometry (Leistritz 276L/32D; 125–170 °C, gradually increasing the temperature from feed to die), and (e) cutting the samples to the desired shape (2 cm × 2 cm; the thickness of tested samples was around 0.05 cm).

### 2.3. Degradation Process

During their respective degradation processes, the samples were permanently placed in a horizontal position at a “shadow-free space” located at Guadalajara City, México (Latitude: 20.7° N, Longitude: 103.3° W). To take advantage of the diversity of the environmental conditions that this geographical position offers throughout the year, the nine degradation periods (P1 to P9; each one with 40 days of duration) described in Table 3 were established. Aside from this, to evaluate the progress of the degradation process occurring in each formulation (F1 to F6; their respective compositions are shown in Table 3), during each degradation period, three samples of each formulation were extracted at each one of the following degradation times: 0, 3, 6, 9, 12, 15, 20, 25, 30, 35, and 40 days.

To describe the environmental conditions occurring throughout each degradation period, the environmental temperature, the index of UV radiation, and the rain level monitored by the Astronomy and Meteorology Institute at the University of Guadalajara were used (this institute permanently reported the these values every 10 min); the data were collected by using a Davis instruments 6163 Wireless Vantage Pro2 Plus with a sensor of UV radiation. The instantaneous values for the environmental temperature (in °C) and incident UV-energy (in kJ/m^2^) were found to be continuously changing along each day and, globally speaking, such values were different each day, but followed, in general terms, a smooth profile.

Thus, to simplify the comparison among the temperature profile throughout of each of the different degradation periods, for each period, a curve representative was built, which was estimated considering only 24 representative values (one for each hour of the day). Each one of these representative values represented the average temperature at the correspondent hour of day, during the respective period; for example: the representative value at 7.5 h (which represented the thermal behavior between 7:00 and 7:59 AM) was the average of the following 240 values (6 values per day, one each 10 min, during the 40 days of the period): day 1 of the period at 7:00, 7:10, 7:20, 7:30, 7:40, and 7:50 AM, day 2 of the period at 7:00, …, 7:50, day 3 of the period at …. day 39 of the period at 7:10, …, 7:50 AM, and day 40 of the period at 7:10 AM, 7:20 AM, 7:30 AM, 7:40 AM, and 7:50 AM.

**Figure 1:**
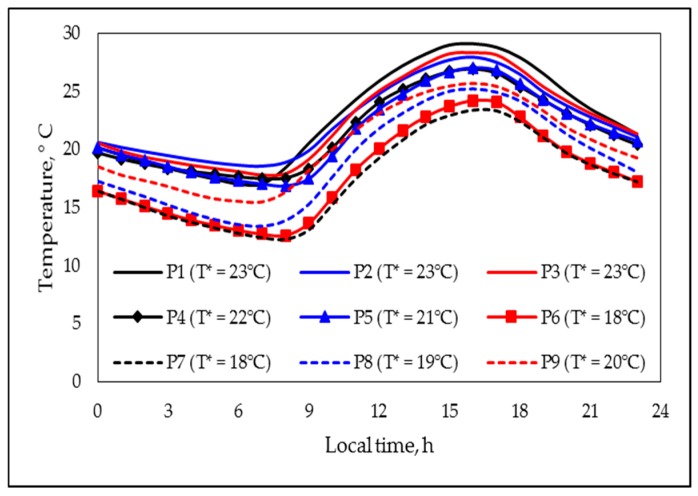
Representative temperature profiles and temperature (T*) values, corresponding to the indicated degradation periods (see codes in image).

Thus, the global thermal behavior during the nine tested degradation periods can be represented by nine curves (one for each degradation period; Figure 1). In Figure 1, it can be noticed that the nine curves displayed quite a similar profile, where few cross-points can be observed, being especially important to note that the pairs curve 1–curve 7 and curve 4–curve 8 did not show any cross point; the temperature effect was studied comparing such pairs. Under these circumstances, when performing qualitative comparisons, an additional simplification can be proposed, representing the behavior of each curve through only one value (T*), which would be obtained by averaging the 24 individual values used to build the correspondent curve. The representative temperature values (T* values) corresponding to each period are also presented in Figure 1.

**Figure 2:**
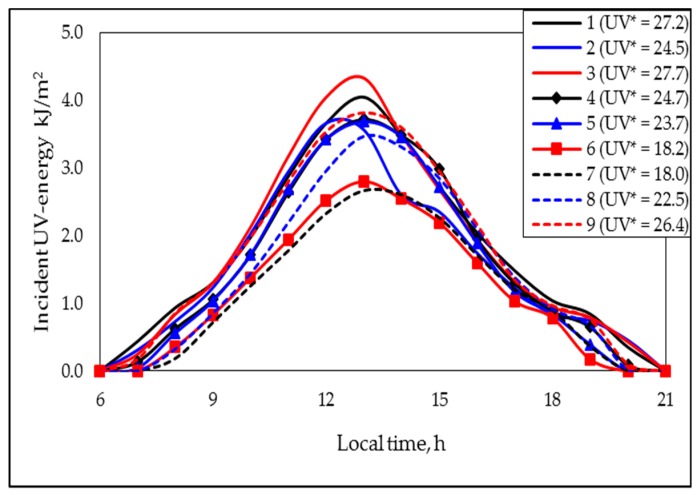
Representative incident UV energy profiles and UV energy (UV*) values, corresponding to the indicated degradation periods (see codes in image; the UV* values were expressed in (kJ)(h)/m^2^).

In an equivalent way, the incident UV-energy values were averaged just as the temperature values were averaged (see procedure above), to obtain the correspondent nine representative curves, which are presented in Figure 2. There it can be noticed that the cross-points’ presence was more common than in Figure 1. However, the cross-points were practically absent when the curves 1, 4, 7, and 8 were compared; with these curves, the combined effect of the simultaneous modification on the T* and UV* values was studied. Therefore, the additional simplification applied to the temperature profile can also be applied to the incident UV energy. In this case, the UV* value was not defined as an average value (such as in the temperature case), but rather was defined as the area under the correspondent curve (note that the UV* index was expressed in (kJ)(h)/m^2^). Thus, Figure 2 also presents the representative incident UV energy values (UV* values).

**Figure 3:**
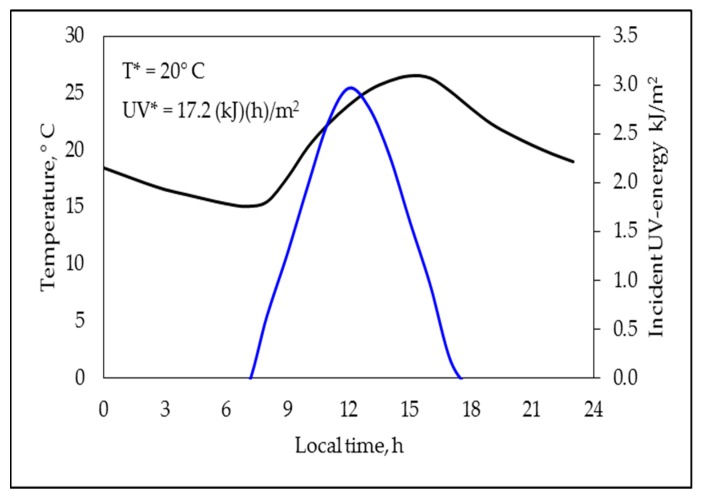
Representative profiles for temperature (black line) and incident UV energy (blue line), as well as the T* and UV* values, corresponding to the “twin experiment”.

To analyze the effect of rain on degrading behavior, the days with rain in each period, as well as the relative amount of rain accumulated during the period (in L/m^2^), were a priori considered as suitable indexes to perform comparisons; such indexes are presented in Table 4. Otherwise, to perform a more suitable estimation of the rain effect, an additional experiment was designed (named the “twin experiment”). In the “twin experiment”, two sets of equivalent samples were simultaneously degraded, as in the previous samples, during the period elapsed between 7 November and 16 December (this period was completely absent of rain); however, the first set of samples was immersed in water (period code: submerged), maintaining a permanent distance between the free surface of the water and any point on the superior surface of the tested samples between 0.5 and 1.0 cm, whereas the other set remained in normal outdoors conditions (period code: not submerged). The profiles along the time of the environmental temperature and the incident UV energy on this experiment were mathematically treated as the equivalent values for the other periods, with Figure 3 presenting the correspondent representative profiles, as well as the T* and UV* values.

### 2.4. Characterization of Degradation

To characterize the degradation process, the PABs of samples were followed by measuring their absorbance with a UV-visible spectrometer (Cintra 6 GBC). The concentration of the different polyenes was quantified, considering the absorbance values measured in the solid samples at the correspondent characteristic wavelengths [32] and reported values for the respective extinction coefficients [32]. Otherwise, to improve the representativeness of the measurements, each spectroscopic characterization was performed in three different but equivalent samples, with the “error bar” considered as representative for such characterization, calculated as follows: $\bar{X}\pm se$, with $\bar{X}$ being the mean value of the three aforementioned measurements, and *se* being the correspondent standard error. 

## 3. Results and Discussion

### 3.1. Evolution of the Concentration of the Different Polyenes (PAB)

To start this section, it is useful to reconsider the set of mechanistic reactions presented in Table 1 and Table 2, noting that the equations for the material balance could be established according to such a set of reactions. Although the mathematical handle of the set of equations for the material balance is difficult, the main problem is that the required reaction constants is not known, which demotivate their use in calculating the evolution of the concentration of the different polyenes through the degradation time. Nevertheless, when the set of equations for the material balance (obtained with the reactions summarized in Table 1 and Table 2) was analyzed in-depth, it was noticed that in these equations there were several positive terms, which anticipated an increment on the respective concentrations as the degrading process advanced; however, there were also other negative ones, which had the opposite effect. In these circumstances, a pseudo-empirical mathematical model could be useful, where the positive terms could be empirically grouped and fitted with a suitable equation, whereas the negative ones could be fitted by other equation.

Thus, for each formulation–degradation system (coded as: FX-PY; 1 ≤ X ≤ 6 and, 1 ≤ Y ≤ 9) there were a set of eleven “error bars”; one for each degradation time at which the concentration of the polyene “*n*” was determined. This set of “error bars” was mathematically fitted by using the following pseudo-empirical equation:

(1) $$P_{n}\left(t\right)=\left(A_{n}e^{t}\right)-\left(c_{n}+\;m_{n}t\right)$$

where the instantaneous concentration of the polyene *n* was estimated considering an exponential accumulation (which empirically grouped all the positive terms of the equation for the material balance of the respective species) and a linear decreasing of the *P_n_* value (for considering the global effect of the negative terms of the respective equation). It is important consider the fact that Equation (1) was defined to introduce the time (*t*) in years.

The parameters in Equation (1) (*A_n_*, *c_n_* and *m_n_*) were numerically estimated. The calculations were performed by using a semi-automatized process helped by a calculation sheet in Excel, which was ex-profeso designed for this objective, where the fitting procedure named “least squares” was adapted to the system. Thus, for each one of the 54 tested formulation–degradation systems, a “family of curves” was obtained (one for each *n* value; 6 ≤ *n ≤* 20). However, since the effect of the *n* value on the evolution of the concentration of the different polyenes was smooth and monotonic, to shorten the information presented, only the results for the *n* pair values are shown below.

**Figure 4:**
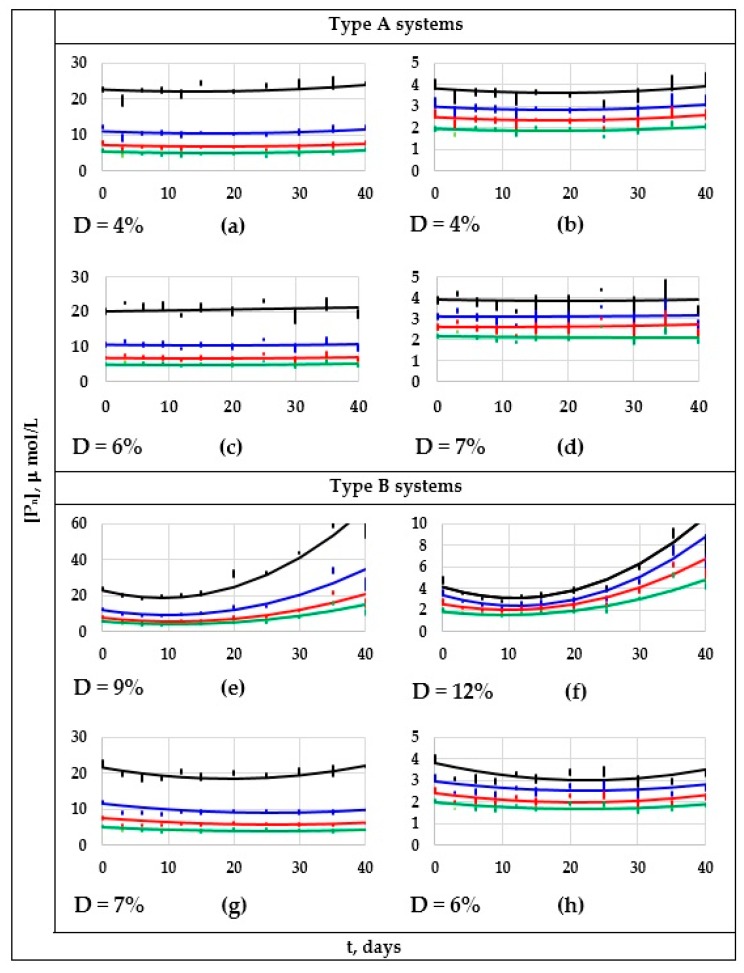
*P_n_* vs. *t*-fitted curves and experimental “error bars” for the four extreme “type A systems” (F1-P6 and F3-P7) or “type B systems” (F1-P1 and F2-P3). “Type A systems”: F1-P6 system (**a**,**b**), and F3-P7 system (**c**,**d**); for (**a**,**c**): *n* = 6 (black lines), 8 (blue lines), 10 (red lines), and 12 (green lines), whereas for (**b**,**d**): *n* = 14 (black lines), 16 (blue lines), 18 (red lines) and, 20 (green lines). “Type B systems”: F1-P1 system (**e**,**f**), and F2-P3 system (**g**,**h**); for (**e**,**g**): *n* = 6 (black lines), 8 (blue lines), 10 (red lines), and 12 (green lines), whereas for (**f**,**h**): *n* = 14 (black lines), 16 (blue lines), 18 (red lines) and, 20 (green lines).

Since the PABs shown by the tested formulation–degradation systems can clearly be grouped by only two types of behavior, Figure 4 only presents the extreme cases of both types. The systems of the first type presented non-significant changes on the concentrations of the different polyenes through the degradation period (“type A systems”), and the systems of the second type presented important changes in these concentrations (“type B systems”). Thus, to exemplify the behavior of the 42 “type A systems”, Figure 4a–d presents the information corresponding to the two extreme “type A systems”, that is, the F1-P6 and F3-P7 systems. Similarly, to exemplify the 12 remaining systems (“type B systems”), the information corresponding to the systems of this type that showed the highest and the lowest degradation level is shown in Figure 4e–h (F1-P1 and F2-P3 systems).

In Figure 4, it can be noticed that, considering the experimental variability expectable on complex systems, the concentrations of the diverse polyenes present on the “type A systems” (Figure 4a–d) remained practically unaltered during their outdoors expositions, being very probable that, at the tested conditions, neither photo-oxidization reactions nor the zipper dehydrochlorination process had been occurring (at least, not to a suitably measurable level). From the available literature [1,5,10,24,27,28,29,30,31], it could be justifiably assumed that bleaching is a generalized phenomenon. However, the presented experimental evidence demonstrate that bleaching is far from being a generalized phenomenon that occurs in any formulation containing PVC (e.g., the bleaching phenomenon was clearly appreciated only in the tested ZnSt_2_-free formulations, or in formulations containing a comparatively low amount of such stabilizer), that was underwent at any outdoor degrading condition (e.g., the bleaching was not experimentally evidenced during the degrading periods 5, 6, and 9). Since all initial samples contained certain amount of each polyene sequence, the absence of degrading reactions during the outdoors exposition (photo-oxidization and/or dehydrochlorination reactions) cannot be explained by the absence of polyene sequences in the system; in fact, formulations F1 or F2 were used to obtain both types of systems. Thus, additional experimental evidence is required to be able to predict the environmental conditions at which a “type A system” can be observed; part of such evidence is now being obtained in our laboratories.

Conversely, in “type B systems”, the degradation process produced an important change on the concentrations of the different polyenes. Therefore, the discussion will furthermore mainly be focused on the “type B systems”. Thus, observing in Figure 4 the D values for this type of system (D is defined as the average percentage deviation among the fitting curves and the correspondent “error bars”), it can be affirmed that such values can be considered low (e.g., on average, lower than 9%), therefore, the proposed fitting equations can be considered as suitable.

Thus, looking for a “master curve” for the “type B systems”, the aforementioned individual curves were normalized with their respective *P_n_*(0) values (initial concentration of the respective polyene *n*), expressing such normalized curves as below:

(2) $${\bar{P}}_{n}\left(t\right)=\;\frac{P_{n}\left(t\right)}{P_{n}\left(0\right)}=\;\frac{\left(A_{n}e^{t}\right)-\left(c_{n}+\;m_{n}t\right)}{\left(A_{n}-\;c_{n}\right)}$$

**Figure 5:**
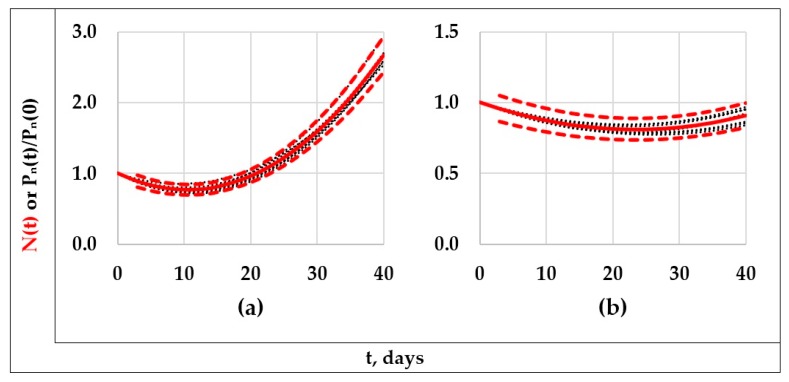
“Master curves” (continuous lines in red) considered as representative for the systems F1-P1 (**a**) and F2-P3 (**b**), their respective limiting curves (dotted-lines in red) denoting a deviation of ±10% with respect to its correspondent “master curve” and the respective $P_{n}\left(t\right)/P_{n}\left(0\right)$ vs. *t* curves (dotted-lines in black).

Although this normalization process was applied to the available 12 “type B systems” (FX-PY systems, with X: 1 or 2, and Y: 1, 2, 3, 4, 7, or 8), obtaining similar results in all these cases, shortening the results presented, only the graphic results corresponding to the formulations F1-P1 and F2-P3 are shown in Figure 5 (note that they are the two extreme “type B systems” considered in Figure 4). In Figure 5, it can be noticed that the fifteen normalized curves corresponding to each formulation–degradation system were very close among them (dotted-lines in black); in fact, all of them can be enclosed inside a narrow area limited by the correspondent dotted-lines in red. The dotted-lines in red were defined to simulate the behavior of a master curve (continuous line in red) that exhibited a hypothetical experimental error of ±10%. On the basis of the information presented in Figure 5, henceforth it will be considered that one unique master curve can suitably represent to the set of original curves.

The respective master curves (one for each one of the 12 “type B systems”) were fitted considering the averages of the values of the respective normalized curves evaluated at the different experimental times (*N(t)* values), which were calculated as indicated below:

(3) $$N\left(t\right)=\;\frac{1}{15}\sum_{n=6}^{20}\left\{{\bar{P}}_{n}\left(t\right)\right\}$$

where ${\bar{P}}_{n}\left(t\right)$ is the value of the curve normalized for the *n*-*esim* polyene, evaluated at the time *t* (t: 0, 3, 6, 9, 12, 15, 20, 25, 30, 35, 40). Note that as a consequence of the normalization process, for all normalized curves ${\bar{P}}_{n}\left(0\right)=1.0$ and, therefore, *N*(0) = 1.0. Consequently, when the fitting of the *N*(*t*) points were performed by using an equation equivalent to Equation (1), the parameters C and A became dependent (in fact, A−C = 1.0), therefore, the new fitting equation could be written as follows:

(4) $$N\left(t\right)=A\left(e^{t}-1\right)-mt+1$$

Note that Equation (4) contains only two fitting parameters, which were obtained following a “least squares” type fitting procedure; the respective parameters A and m are presented in Table 5. To warrant that the master curve can be mathematically reproduced with an inaccuracy level lower than 0.1%, the parameters A and m were presented in Table 5, using five significant figures.

Additionally, to re-calculate the fifteen curves that correspond to each of the different polyenes (6 ≤ *n* ≤ 20), it is required that the respective initial polyene concentrations ($P_{n}\left(0\right)$values) are available; in this work, the next fitting function was used:

(5) $$P_{n}\left(0\right)=\left(R\right)e^{\left(\left(S\right)\left(n\right)\right)}+\left(U\right)e^{\left(\left(V\right)\left(n\right)\right)}$$

Therefore, by using Equations (4) and (5), the PAB through the different degradation periods can be recalculated, with the respective R, S, U and V fitting parameters being also included in Table 5.

Particularly for comparative purposes, there is an evident advantage when describing the PAB in a given system by using one unique curve (“master curve”) instead of a family of curves. This advantage has also been exploited when the Yellowness index (YI) has been used to describe the degrading behavior of different samples [33,34,35]. Unfortunately, there was not any numerical relationship correlating the YI of a given sample with the concentrations of each one of the different polyenes contained in it; in fact, it is very probable that there is not a univocal relationship among a given YI value and the different *P_n_* values. Contrarily, the proposed master curve allowed us to calculate the evolution of the concentrations of the different polyenes as the degradation time increased (central objective of this work), which represented a relevant advantage when compared to the YI values. Alternatively, although the PAB can also be calculated using the complete set of equations for the material balance (the set of equations obtainable considering the reactions included in Table 1 and Table 2; “alternative procedure”), it was made evident the considerably higher difficulty (and maybe the practical impossibility) involved in this “alternative procedure”.

**Figure 6:**
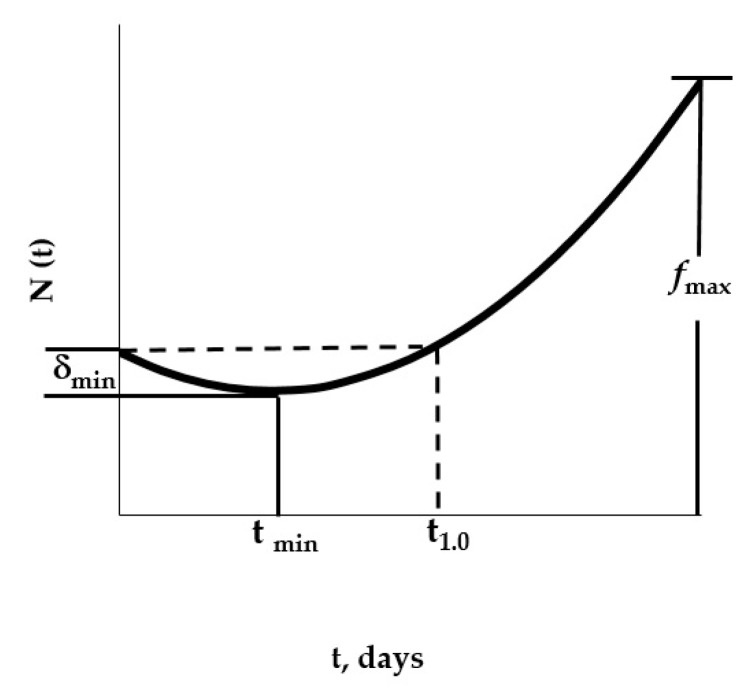
Characteristic values (*δ_min_*, *t_min_*, *t*_1.0_, and *f_max_*) for the respective master curves.

When the respective parameters A and m presented in Table 5 were used in Equation (4), the respective “master curves” were reproduced, and the respective values for *δ_min_*, *t_min_*, *t*_1.0_, and *f_max_* characterizing to each “master curve” were obtained; such values are graphically defined in Figure 6 and numerically presented in Table 5.

Thus, when comparing the *δ_min_* values for the different “type B systems” (Table 5), it is clear that, in all cases, the bleaching phenomenon occurred, producing a noticeable decrease on the different polyene concentrations that were usually around 0.24.

This phenomenon allows for the obtaining of samples that present a color less intensive than those observed at *t* = 0 (at least up to *t*_1.0_); this phenomenon is especially relevant in plasticized, transparent and lacking of artificial color materials, such as those here considered. Thus, when the lifetime of this type of samples is expectably lower than the *t*_1.0_ value, this phenomenon could be considered technologically beneficial. The values of *t_min_*, *t*_1.0_, and *f_max_* are also presented in Table 5. There, it can be observed that as the *f_max_* values decreased, the *t*_1.0_ value increased, generating a comparatively higher time interval where the bleaching phenomenon could be technologically useful. Note that, at post-processing times between 0 and *t_min_*, an increment on the post-processing time resulted in a decrement on the sample color intensity, however, at times higher than *t_min_*, the effect was the opposite; therefore, it is important to know the *t*_1.0_ values, as well as the *t_min_* values, characterizing to each “type B system”. To present a more in-depth analysis, it can be noticed in Table 5 that, inclusive of the formulation F1 (which exhibited the highest *f_max_* values), when it was degraded at P1 or P2 conditions, there were around 3 weeks where it was possible take technological advantage of the bleaching phenomenon. However, at the degrading conditions coded as P4, such a formulation allowed for the exploiting of such advantages during a noticeably longer period (around 6 weeks). In addition, the formulation F2, during the majority of the year (excepting during the period P1), exhibited *t*_1.0_ values larger than 6 weeks, which was a time interval that could be useful in several practical scenarios.

### 3.2. The Degradation Period Effect

**Figure 7:**
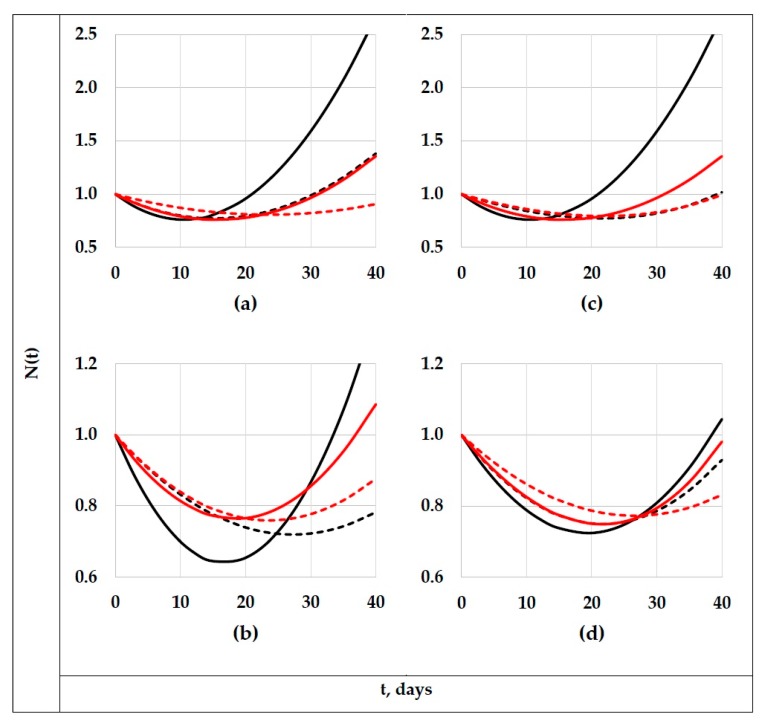
Graphic representation of Equation (4) (“master curves” N vs. *t*), considering the respective parameters indicated in Table 5 for the formulations F1 (lines in black) and F2 (lines in red) degraded during the following periods: (**a**) P1 (continuous lines) and P3 (dotted-lines); (**b**) P_not submerged_ (continuous lines) and P_submerged_ (dotted-lines); (**c**) P1 (continuous lines) and P7 (dotted-lines); (**d**) P8 (continuous lines) and P4 (dotted-lines).

Up to now, the individual PAB showed for the tested formulations that underwent different degrading conditions has been described in a generalized way. However, a comparative analysis of the effect on the PAB produced by the respective degrading process has not been discussed yet. Thus, for comparative purposes, in Figure 7, the “master curves” representing the PABs showed by several “type B systems” are presented. Figure 7a presents the behaviors shown for formulations F1 (black lines) and F2 (red lines) degraded during the periods P1 (continuous lines; representative parameters: T*: 23 °C (Figure 1); UV*: 27.2 (kJ)(h)/m^2^ (Figure 2); days with rain: 0 (Table 4)) and P3 (dotted lines; representative parameters: T*: 23 °C (Figure 1); UV*: 27.7 (kJ)(h)/m^2^ (Figure 2), days with rain: 23 (Table 4)). Herein, it can be noticed that the rain produced a considerable decrease on the *f_max_* value jointed with a slight/very slight decrease in *δ_min_* values, as well as a noticeable increment in the *t_min_* value, with the respective numerical values being presented in Table 5. However, a more in-depth analysis considering the estimated values of the fitting parameters (A and m values presented in Table 5) allowed us to affirm that there was a diminution on the global rate of “production of polyenes” (the A values for the systems with rain (period P3) were lower than those for the “dry systems” (period P1)), but also on the global rate of “consumption of polyenes” (the rain presence also produced decrements in the correspondent m values). Nonetheless, the PAB shown for these systems demonstrated that, at short times (<*t*_1.0_), the global effect was dominated by the photo-oxidation reactions, yet, at longer times, the dominating process was the zipper dehydrochlorination. Thus, it can be affirmed that the rain produced a “generalized stabilizing” effect, which, at least as a first hypothesis, can be attributed, among other factors, to (a) the partial reflection of the UV light [36] produced by the free surface of the rain drops deposited on the horizontally positioned polymer surface (it would be expected that this fact slow down the rate of photo-oxidation reactions), (b) the physical barrier to the oxygen transfer produced by the presence of multiple drops on the polymer surface (this fact also decreased the rate of photo-oxidation reactions [31]), and (c) the “cooling effect” produced by the presence and eventual evaporation process of the drops (the drop presence favored the polymer temperature being lower than the environmental temperature, which mainly promoted a decrement on the rate of the dehydrochlorination process).

When comparing the PABs during the periods P1 and P3 (note that the UV* and T* values were quite similar during such periods), the effect of the rain could be considered as practically decoupled. Nevertheless, to confirm this assumption, a “twin experiment” was designed and performed under the conditions of the periods coded as P_submerged_ and P_not submerged_ (additional information is presented in Table 3 and Figure 3). Qualitatively speaking, the Figure 7a (comparative analysis between the periods P1 and P3) and 7b (comparative analysis between periods P_submerged_ and P_not sumerged_) exhibited the same general trends, that is, the submerged formulations (and those considered in the period P3) presented lower values of m and A than their not submerged equivalents (and those considered in the period P1). Nonetheless, in the “twin experiment” it was observed that the immersion (used to simulate the rain in a controlled way) also promoted an important decrease on the *f_max_* and *δ_min_* values and an increment on the *t_min_* value, which allowed confirming the stabilizing effect of the rain.

The combined effect of a decrement on the T* and UV* values can be estimated considering the information presented in Figure 7c; usually, under the tested environmental conditions, there was a direct relation between the respective T* and UV* indexes. In the figure, the results obtained with the formulations F1 and F2 degraded at the conditions P1 (continuous lines; representative parameters: T*: 23 °C (Figure 1); UV*: 27.2 (kJ) (h)/m^2^ (Figure 2)) and P7 (dotted lines; representative parameters: T*: 18 °C (Figure 1); UV*: 18.0 (kJ) (h)/m^2^ (Figure 2)) are presented. Since the period P1 was, in general terms, the warmest in the considered year, and P7, the coolest one, this comparison can be considered as extreme, at least in Guadalajara City, having the additional benefit that the rain was absent in both periods. In Figure 7c, it can be noticed that, as would be expected, the decrement on the T* and UV* values produced a stabilizing effect on the polymer formulations, that is, produced an important decrement on the *f_max_* value jointed with a slight/very slight diminution on the *δ_min_* value, as well as a considerable increment on the *t_min_* value; the numerical values are presented in Table 5. Mathematically speaking, as occurred in systems considered for estimating the “rain effect”, the values of the A and m parameters were importantly diminished by the change in the environmental conditions, in this case, by the decrement in the respective indexes T* and UV*. Thus, for these systems, it could be expected that the decrement on the T* index decreased the rate of the thermically favored reactions, that is, the dehydrochlorination reactions (they were globally simulated thorough the A parameter; the values of this parameter were lower for systems degraded during the period P7 as compared with those one degraded during period P1). Complementarily, the decrement on the UV* index decreased the rate of the photo-oxidation reactions (which were globally simulated thorough the m parameter; this parameter behaved as the A one).

Finally, the relative importance of the opposite effects produced by the rain presence and the global action involved in the simultaneous increment on the indexes UV* and T* could be estimated by comparing the PABs shown by formulations F1 and F2, degraded during the periods P8 (continuous lines; representative parameters: T*: 19 °C (Figure 1); UV*: 22.5 (kJ) (h)/m^2^ (Figure 2), days with rain: 0 (Table 4)) and P4 (dotted lines; representative parameters: T*: 22 °C (Figure 1), UV*: 24.7 (kJ) (h)/m^2^ (Figure 2), days with rain: 27 (Table 4)); the results for such formulation–degradation systems are shown in Figure 7d. There, the stabilizing effect of the rain can be confirmed, which produced a decrement on the *f_max_* and *δ_min_* values and an increment on the *t_min_* value. However, it was clear that the “rain effect” was partially countered by the effect of the increment in the T* and UV* values produced during the period P4 (when it was compared with the period P8). Thus, although in this comparison the “rain effect” was the dominant effect (formulations degraded during the period with rain (period P4) were less altered by the degrading procedure), the combined effect of temperature and UV irradiation was also important; this became evident when the differences between the PABs produced during the periods P4 and P8 were compared with those produced during the analysis of the “individual rain effect” or the “combined UV*–T* effect”.

An integral analysis of the last three effects allowed us to affirm the qualitative agreement among them, but also allowed us to estimate from experimental data the relative importance of the “rain presence” and the increment on the UV* and T* values, which is information that could be qualitatively useful when other experimental systems are being studied.

## 4. Conclusions

The bleaching phenomenon was hereby quantitatively studied, demonstrating that, in the most of studied systems (“type A systems”), this phenomenon did not occur or, at least, did not occur at a measurable level, which demonstrated that this is not a generalized phenomenon that occurs at any formulation containing PVC exposed in outdoor conditions, as it could a priori be inferred from the literature [1,5,10,24,27,28,29,30,31]. However, when the bleaching occurred (“type B systems”), it produced an important diminution on the amount of polyenes accumulated in samples, which represented a usable technological advantage, especially in the case of plasticized, transparent and lacking of artificial color samples, such as those considered in this study. Thus, as part of this work, a general methodology to define environmental indexes was proposed (T*, UV*, and days with rain), which allowed for globalizing, in a suitable way, the variable environmental conditions occurring throughout the considered degrading periods. Additionally, a mathematical procedure to obtain an general equation that describes the PAB on the “type B systems” was proposed, which required only two primary fitting parameters (A and m) and four secondary fitting parameters (R, S, U, V). Moreover, the stabilizing effects of the rain presence and the combined decrement on the T* and UV* indexes were experimentally quantified. Finally, the fitting equation proposed here and the respective parameter values were suitably interpreted and used to estimate the relative importance of the thermally favored reactions (e.g., dehydrochlorination reactions), with respect to the photo-oxidative reactions simultaneously occurring in the studied systems.

## Figures and Tables

**Table 1:** Degrading reactions that affect the polyene accumulation behavior on poly(vinyl chloride) (PVC) formulations being photo-thermally oxidized.

	Reactions	PVC	PVCOOH	PVC•	PVC•_(frac)_	PVCOO•	P_1(frac)_	P_1_	P_a_	P_b_	P_c_	P_n_	P_m_	P_s_	P_t_	P^c^_n-2_	P_n+1(frac)_	P_n+1_	P_n_OOH	P_1_•	P_n_•	P_n+1_•	P_n_OO•	O_2_	HCl	Cl•
PF	a)			R				P																		P
	b)^*^			R	P		P																			
PI	c)																	P			R				P	R
	d)																	P			R					P
	e)				P												P				R					
PD	f)^+^								P	P	P	R	R													
	g)^*^											R		P	P											
	h)	R		P												P							R			
OR	i)	R		P																						P
	j)											R									P					P
	k)	R		P																					P	R
	l)							R												P					P	R
	m)																	R				P			P	R
	n)																				R		P	R		
	o)	R		P															P				R			
	p)			R		P																		R		
	q)	R	P	P		R																				
	r)	R		P								P									R					
PVC 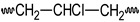						PVCOOH 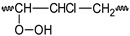							PVC• 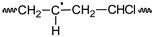							PVCOO• 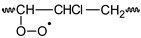						
P_n_ 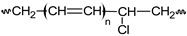 n ∈ {a,b,c,n,m,s,t,1}						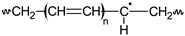 P_n_• n ∈ {a,b,c,n,m,s,t,1}							P_n_OOH 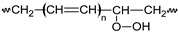							P_n_OO• 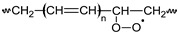						

R: denotes the reactants and P denotes the products. ^+^ Since the formed branched molecule (P_a,b,c_^bran^) contains three independent polyene sequences, for polyene balance, it is equivalent to three individual molecules containing each one in a polyene sequence (P_a_ + P_b_ +P_c_ ≡ P_a,b,c_^bran^); see P_a,b,c_^bran^ structure in the footnote in Table 2. * Since the formed branched molecule (P_s,t_^bran^) contains two independent polyene sequences, for polyene balance, it is equivalent to two individual molecules containing in each one a polyene sequence, therefore, (P_s_ + P_t_ ≡ P_s,t_^bran^); see P_s,t_^bran^ structure in the footnote in Table 2. PF: polyene formation, PI: polyenic increasing, PD: polyene decreasing, OR: other reactions.

**Table 2:** Degrading reactions that affect the polyene accumulation behavior and the molecular weight distribution on PVC formulations being photo-thermally oxidized.

	Reactions	PVC•	PVC•_(frac)_	P_1(frac)_	P_a,b,c_^bran^	P_n_	P_m_	P_s,t_^bran^	P_n+1(frac)_	P_n_•
**PF**	(a)	R	P	P						
**PI**	(b)		P						P	R
**PD**	(c) ^+^				P	R	R			
	(d) *					R		P		
PVC• 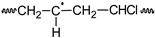				P_n_ 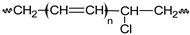 n ∈ {n,m,1}			P_n_• 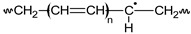 n ∈ {n,m,1}			
P_s,t_^bran^ 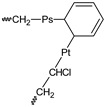				P_a,b,c_^bran^ 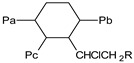						

R: denotes the reactants and P denotes to products. ^+^ Note that this reaction is the same as that in f) in Table 1, but in this table it is schematized (see the last line in this Table) and coded (P_a,b,c_^bran^) as a branched molecule, denoting that, besides modifying the polyene accumulation behavior, it also modifies the molecular weight distribution. * Note that this reaction is the same as that in g) in Table 1, but in this table it is schematized (see the last line in this Table) and coded (P_s,t_^bran^) as a branched molecule, denoting that, besides modifying the polyene accumulation behavior, it also modifies the molecular weight distribution.

**Table 3:** Description of the tested formulations and the considered degradation periods. DEHP: di(2-ethylhexyl) phthalate, ESO: epoxidized soybean oil, CaSt_2_: calcium stearate, ZnSt_2_: zinc stearate.

Formulations		Degradation Periods	
Formulation Code	Relative Content of Components, g/100 g of PVC (PVC/DEHP/ESO/CaSt_2_/ZnSt_2_)	Period Code	Initial Date–Final Date
F1	100/45/3/1.0/0.0	P1	16 April–25 May
F2	100/45/3/0.8/0.2	P2	27 May–5 July
F3	100/45/3/0.6/0.4	P3	7 July–15 August
F4	100/45/3/0.4/0.6	P4	17 August–25 September
F5	100/45/3/0.2/0.8	P5	27 September–5 November
F6	100/45/3/0.0/1.0	P6	7 November–16 December
		P7	18 December–26 January
		P8	28 January–8 March
		P9	10 March–18 April
		submerged	7 November–16 December
		not submerged	7 November–16 December

**Table 4:** Days with rain and L/m^2^ collected in the indicated period.

	P1	P2	P3	P4	P5	P6	P7	P8	P9
Days with rain	0	18	23	27	13	2	0	0	7
L/m^2^ collected in period	0	151	244	324	126	12	0	0	117

**Table 5:** Diverse parameters describing to the indicated master curves and their respective derived parameters.

	**F1-P1**	**F1-P2**	**F1-P3**	**F1-P4**	**F1-P7**	**F1-P8**	**F1-P_submerged_**	**F1-P_not submerged_**
*m*	567.79	487.46	261.41	144.15	156.81	198.40	103.08	340.00
*A*	551.64	472.66	250.63	135.74	148.53	188.11	95.655	324.63
*δ_min_*	0.23	0.23	0.23	0.26	0.23	0.28	0.28	0.36
*t_min_*	11	11	15	22	20	19	27	17
*f_max_*	2.67	2.32	1.38	0.92	1.02	1.04	0.78	1.34
*t*_1.0_	21	22	30	>40	39	38	>40	33
*R*	27.34	29.94	16.89	31.58	30.82	28.39	25.27	24.05
*S*	−0.1328	−0.1254	−0.1074	−0.1336	−0.1351	−0.1289	−0.1215	−0.1203
*U*	700.5	1645	417.0	687.2	1856	1709	939.1	702.2
*V*	−0.7002	−0.8151	−0.5441	−0.7095	−0.8593	−0.8349	−0.7305	−0.6727
	**F2-P1**	**F2-P2**	**F2-P3**	**F2-P4**	**F2-P7**	**F2-P8**	**F2-P_submerged_**	**F2-P_not submerged_**
*m*	263.41	90.681	95.031	87.472	133.49	160.32	81.943	184.40
*A*	252.31	83.886	89.109	81.303	126.24	151.53	76.465	175.22
*δ_min_*	0.24	0.27	0.19	0.23	0.20	0.25	0.19	0.24
*t_min_*	16	28	23	27	20	21	25	19
*f_max_*	1.36	0.78	0.91	0.83	0.99	0.98	0.88	1.09
*t*_1.0_	31	>40	>40	>40	>40	>40	>40	37
*R*	21.35	15.68	16.67	15.39	21.99	20.83	17.37	20.83
*S*	−0.1204	−0.1034	−0.1070	−0.1026	−0.1192	−0.1184	−0.1072	−0.1184
*U*	484.0	262.4	274.2	236.1	1087	525.9	277.6	525.4
*V*	−0.6189	−0.5005	−0.5114	−0.4865	−0.7448	−0.6245	−0.5143	−0.6284
